# Sucrosomial Iron Supplementation in Anemic Patients with Celiac Disease Not Tolerating Oral Ferrous Sulfate: A Prospective Study

**DOI:** 10.3390/nu10030330

**Published:** 2018-03-09

**Authors:** Luca Elli, Francesca Ferretti, Federica Branchi, Carolina Tomba, Vincenza Lombardo, Alice Scricciolo, Luisa Doneda, Leda Roncoroni

**Affiliations:** 1 Center for the Prevention and Diagnosis of Celiac Disease, Gastroenterology and Endoscopy Unit, Fondazione IRCCS Ca’ Granda Ospedale Maggiore Policlinico, 20122 Milan, Italy; tomba.carolina@gmail.com (C.T.); vincenza.lombardo@policlinico.mi.it (V.L.); alice.scricciolo@policlinico.mi.it (A.S.); leda.roncoroni@tiscali.it (L.R.); 2 Department of Pathophysiology and Transplantation, University of Milano, 20100 Milan, Italy; francesca.ferretti@unimi.it (F.F.); federica.branchi@unimi.it (F.B.); 3 Department of Biomedical, Surgical and Dental Sciences, University of Milano, 20100 Milan, Italy; luisa.doneda@unimi.it

**Keywords:** sucrosomial iron, iron sulfate, celiac disease, iron deficiency anemia

## Abstract

Patients with celiac disease (CD) frequently suffer from iron deficiency anemia (IDA) and may benefit from iron supplementation. However, intolerance to iron sulfate and duodenal atrophy could reduce the efficacy of this supplementation. This study evaluated the efficacy of a new sucrosomial iron formulation in patients with CD. Consecutive patients with CD and IDA were divided into two groups: patients with a known intolerance to iron sulfate were treated with sucrosomial iron (30 mg of iron/day), while those receiving iron supplementation for the first time were assigned to iron sulfate (105 mg of iron/day). Forty-three patients were enrolled (38 females, mean age 49 ± 9 years). After a follow-up of 90 days both groups showed an increase in Hb levels compared to baseline (+10.1% and +16.2% for sucrosomial and sulfate groups, respectively), and a significant improvement in all iron parameters, with no statistical difference between the two groups. Patients treated with sucrosomial iron reported a lower severity of abdominal symptoms, such as abdominal and epigastric pain, abdominal bloating, and constipation, and a higher increase in general well-being (+33% vs. +21%) compared to the iron sulfate group. Sucrosomial iron can be effective in providing iron supplementation in difficult-to-treat populations, such as patients with CD, IDA, and known intolerance to iron sulfate.

## 1. Introduction

Celiac disease (CD) is a common autoimmune disease of the small intestine (SI), with a worldwide prevalence of 1% in Western countries [1,2,3,4,5,6,7]. Notably, patients with CD frequently present with iron deficiency anemia (IDA), which usually reverts with adoption of a gluten-free diet (GFD) and subsequent normalization of the SI mucosa [8,9]. However, some patients present persistent IDA despite GFD. A number of factors may contribute to this scenario, such as persistence of a negative balance between iron intake and loss, persistence of villous atrophy despite correct GFD, or presence of a genetic background that reduces iron absorption [10]. These subjects may benefit from iron supplementation, although both the SI damage and the low tolerance to iron sulfate could reduce its efficacy [11]. Response to iron supplementation could be enhanced by using a tolerated iron form or an iron formulation able to increase intestinal absorption through the intestinal barrier, thus overcoming mucosal atrophy [12]. Noteworthy, a recent consensus paper states that new oral iron products may be efficacious in patients who are intolerant or non-responsive to conventional iron salts, and in those with contraindications to intravenous (IV) iron [13].

Sucrosomial iron (Sideral^®^ Forte, Pharmanutra, Pisa, Italy) is a preparation of ferric pyrophosphate covered by a phospholipids and sucrester membrane, which proved to be effective in the nutritional supplementation of oncological patients, in subjects affected from chronic kidney disease, in pregnant women, and after bariatric surgery [13,14,15,16,17,18,19]. This new formulation shows high gastrointestinal absorption and bioavailability, with a low incidence of side effects thanks to the absence of a direct contact with the intestinal mucosa [14]. Indeed, this formulation consists of a low, non-toxic dose of sucresters, which protect iron from the acid environment of the stomach and increase its permeability, thus allowing higher absorption rates [13,14]. In addition, sucrosomial iron can be absorbed across intestinal epithelium by an alternative route, non-mediated by the DMT-1 carrier [20], which may contribute to the reduction of side effects and the prevention of iron instability in the gastrointestinal tract [20]. Therefore, sucrosomial iron might represent a promising new strategy of iron replacement in patients with CD, but evidence supporting its use in this setting is required. In particular, this nutritional supplementation may have a role in patients with CD with damaged intestinal mucosa, who are often intolerant to standard iron formulations and, therefore, may need to interrupt this supplementation [12].

This prospective study investigates nutritional supplementation with sucrosomial iron in patients with CD and IDA, and a previous therapeutic failure with oral iron sulfate supplementation.

## 2. Patients and Methods

### 2.1. Study Setting and Design

This was a prospective monocentric study, conducted at the Center for Prevention and Diagnosis of Celiac Disease of the Fondazione IRCCS Ca’ Granda Milan, Italy. All patients, enrolled from October 2014 to April 2016, signed an informed consent form before enrolment and the local ethics committee approved the study design (protocol number 1892). The trial has been registered on ClinicalTrials.gov, number NCT02916654.

Consecutive adult patients with treated CD and concomitant IDA, according to current guidelines on CD diagnosis and IDA [21], were eligible to this study. IDA diagnosis was established based on laboratory data, according to Beutler and Waalen [22], i.e., hemoglobin (Hb) < 12.2 g/dL in women and <13.7 g/dL in men. IDA was identified on the basis of serum iron, ferritin < 20 ng/mL and transferrin saturation < 20%. Other types of anemia, as related to folic acid or B12 vitamin deficiency, were excluded to avoid a confounding effect. All patients followed a strict GFD for at least 12 months with normalization of anti-transglutaminase IgA. Patients with a B12 vitamin or folic acid deficiency, reporting a voluntary ingestion of gluten-containing food, with persistence of circulating anti transglutaminase IgA, suggesting gluten ingestion, or with hematological diseases were excluded from the study. When indicated, patients underwent a second duodenal biopsy (at least four oriented duodenal specimens) or other endoscopic investigations, such as capsule endoscopy or colonoscopy.

Consecutive eligible patients were assigned to either ferrous sulfate or sucrosomial iron treatment depending on their previously reported tolerance to ferrous sulfate. The first group, reporting a previous intolerance to ferrous sulfate leading to a suspension of therapy, was treated with sucrosomial iron (Sideral^®^ Forte, Pharmantra SpA, Pisa, Italy), 30 mg of elemental iron per day; the second group was composed by patients tolerant to ferrous sulfate or naïve to iron supplementation who were administered iron sulfate tablets (Ferrograd^®^, Teofarma, Pavia, Italy), 105 mg of elemental iron per day. We decided to use two different doses to counterbalance the higher absorption rate of sucrosomial formulation, as reported by Fabiano et al. in a recent comparative study on in vivo and ex vivo absorption of different iron formulations [20]. Patients’ clinical and demographic characteristics, including duration of GFD and duodenal histology (graded by means of the Marsh-Oberhuber scale [23]), when available, were evaluated. All patients were followed for a period of 90 days, during which three visits comprehensive of blood tests were performed: at baseline (T0), at 45 days (T45), and at 90 days (T90). Therapy compliance was verified during the clinical visits by an interview with the physician.

### 2.2. Evaluations

The following serological parameters were measured to evaluate the iron status: hemoglobin (Hb, g/dL), mean corpuscular volume (MCV, fL), hematocrit (%) (Sysmex XN-9000 automated hematology analyzer, Sysmex, Kobe, Japan), iron (Quantitative ELISA, Roche, Basilea, Switzerland, expressed as µg/dL), ferritin (chemiluminescent method expressed as ng/mL), quantitative determination of transferrin (mg/dL) and transferring saturation calculated following the formula (iron (transferrin × 1.42) × 100). Iron parameters were measured at T0, T45, and T90.

The severity of possible symptoms associated with both treatments, assessed by a 10 cm visual analogic scale (VAS) evaluating diarrhea, constipation, epigastric and abdominal pain, stool consistency, and general well-being were also measured at T0, T45, and T90.

Smoking habits, menstrual losses, and *Helicobacter pylori* infection (13C urea breath test) were also evaluated. All the evaluations were performed directly by the physicians during baseline and follow-up visits and through the administration of questionnaires.

An analysis of Hb levels by stratifying patients according to their Marsh grade (0–2 vs. 3a, 3b, 3c, i.e., duodenal normotrophic mucosa vs. duodenal atrophy) was also performed.

### 2.3. Statistical Analysis

All data were analyzed by descriptive statistics and presented as mean ± standard deviation (SD) and a *p*-value < 0.05 was considered statistically significant. Kolmogorov-Smirnov’s test was used to assess the normal distribution of data. Intra- and inter-group comparisons were performed by the chi-square test or the Fisher’s exact test, as appropriate for discrete variables, and by the Student’s *t*-test or the Mann-Whitney test, as appropriate for continuous variables. Two-way ANOVA was used for variance analysis and further non-parametric tests included Friedman and Wilcoxon tests were performed. Statistical significance was further confirmed using Tukey’s test or Mann-Whitney. 

Statistical analysis was performed by the SPSS software (version 19, IBM, Armonk, NY, USA).

## 3. Results

### 3.1. Patient Populations

In total, 43 patients were enrolled (38 females, age at enrolment 41 ± 10 years, age at CD diagnosis 35 ± 11 years, years on a GFD 8 ± 7 years). Of these, 24 (55%) reported previous side effects and intolerance to iron sulfate and, thus, received sucrosomial iron. In the sucrosomial iron group, nine patients (32%) had an atrophic lesion at duodenal histology (Marsh grade 3a, b or c), as compared to 6 subjects (41%) in the iron sulfate group (Table 1). In addition to CD, in the sucrosomial iron group 1 patient presented autoimmune hepatitis, 1 presented Sjogren disease, and 7 presented Hashimoto thyroiditis; in the iron sulfate group, 6 patients had Hashimoto thyroiditis.

### 3.2. Iron Parameters

Table 2 summarizes iron parameters measured in the two groups at the beginning and at the end of the iron supplementation. At T0 the enrolled CD cohort presented an Hb of 10.9 ± 0.7 g/dL for the sucrosomial iron group, and Hb of 11.0 ± 0.7 g/dL for the iron sulfate group (Table 2). A mean Hb increase versus baseline of 10.1% and 16.2%, was obtained for the sucrosomial iron and iron sulfate group, respectively (*p* = 0.002). In total, 7 patients receiving sucrosomial iron (30%) and 10 on iron sulfate (60%) showed a >15% increase in Hb concentration at T90, compared to T0 (*p* = 0.2). While all patients had abnormal Hb values at baseline, at T90, a similar proportion of patients in the two groups presented Hb values within the range of normality (70% vs. 82%, *p* = n.s.). These results were obtained despite a 33% lower dose of sucrosomial iron compared to iron sulfate.

All other iron parameters significantly improved with both treatments, compared to baseline values (Table 2). No significant differences between groups were reported in any parameter, with the exception of ferritin, which was higher in patients on iron sulfate (Table 2). In both groups, at T90, most patients presented normalized values of all iron parameters (Table 2).

Notably, these results were not influenced by excessive supplementation or blood loss during the course of the study: no transfusions nor other blood supplements were administered to patients during the study, while blood loss in the female population was assessed by measuring the mean number of days of the menstrual cycle (see Table 1).

### 3.3. Effects in Patients with Mucosal Damage

Overall, Hb concentration similarly increased in patients with MARSH grade 0–2 and in those with grade 3a-b-c (MARSH 0–2: 11.2 ± 0.8 g/dL at baseline vs. 12.5 ± 1.0 g/dL at T90, *p* = 0.0001; MARSH 3a-b-c: 10.4 ± 0.7 g/dL at baseline vs. 11.9 ± 0.9 g/dL at T90, *p* = 0.001). 

**Figure 1:**
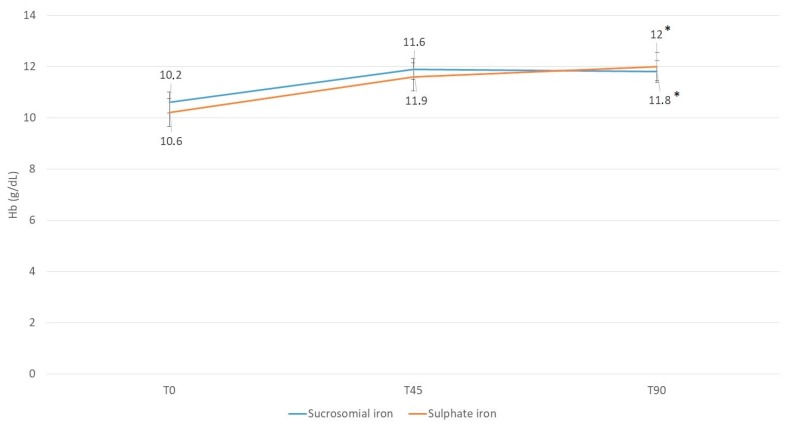
Hb concentration throughout the study period in the two treatment groups in patients with duodenal atrophy (Marsh 3 lesions). * *p* < 0.05 time effect at T90 vs. T0 for both groups. All values are means ± SD.

In patients with MARSH grade 3a-b-c, a similar increase in Hb concentration at T90 was reported in the two groups compared to baseline, despite the use of sucrosomial iron of 1/3 of the iron sulfate dose (sucrosomial iron: from 10.6 ± 0.6 g/dL at T0 to 11.8 ± 0.9 g/dL at T90, +11.1%, *p* = 0.15; iron sulfate: from 10.2 ± 0.9 g/dL at T0 to 12.0 ± 0.9 g/dL at T90, +18%, *p* = 0.03). No difference was found between the Hb concentrations at T90 of patients treated with sucrosomial iron vs. iron sulfate and showing a MARSH grade 3a-b-c duodenal lesion (11.8 ± 0.9 g/dL vs. 12.0 ± 0.9 g/dL, *p* = 0.28) (Figure 1).

### 3.4. Symptoms and Tolerability

Overall, four patients from the enrolled cohort reported an adverse event: 2 patients at T45 (abdominal colic in the sucrosomial iron group and nausea in the iron sulfate group) and 2 at T90 (vaginal itching in the sucrosomial iron group and dyspepsia plus gastro-esophageal reflux in the iron sulfate group). Both adverse events in the iron sulfate group were judged as potentially correlated with treatment; conversely both events in the sucrosomial iron group were considered unrelated to treatment.

**Figure 2:**
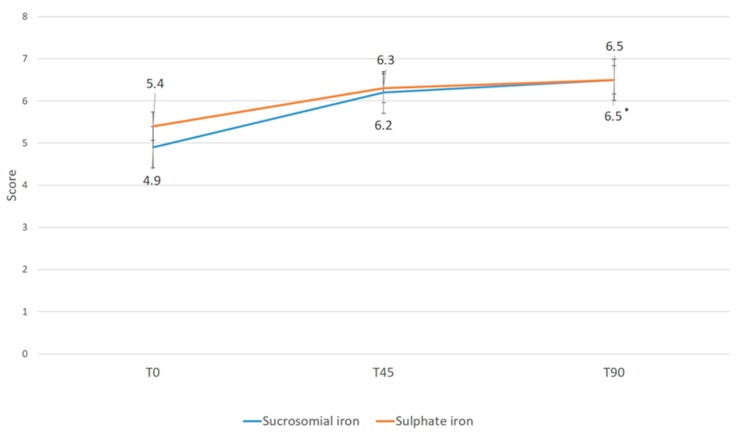
General well-being throughout the study period. All values are mean ± SD. * *p* < 0.05 vs. T0.

Table 3 summarizes the results of symptoms assessment of the two groups throughout the study period. Overall, no significant differences between groups were reported at any time-point, although a lower severity of abdominal and epigastric pain, abdominal bloating, and constipation with sucrosomial iron was reported. Similarly, a greater general well-being was reported with sucrosomial iron, compared to iron sulfate, with similar values in the two groups at T90 (% variation at T90 vs. baseline: +33% vs. +21%; Figure 2).

Two patients in the sucrosomial iron group (9%) and 4 treated with iron sulfate (23%) prematurely discontinued therapy. In the sucrosomial iron group discontinuation was due to adverse events for 1 patient and to patient’s decision for the other, while in the iron sulfate group discontinuation was due to poor efficacy (*n* = 2), adverse events, and patient’s decision (*n* = 1 each).

## 4. Discussion

Iron supplementation is frequently required in patients with CD, who often present poor iron absorption due to intestinal disease [11]. To this end, oral iron formulations are often preferred over IV formulations, due to increased convenience of use and patients’ preference [13]. However, absorption of standard oral iron formulation may be poor in patients with CD due to intestinal malabsorption [13] and they can frequently present side effects, such as abdominal pain, bloating, diarrhea, and constipation.

In this study, conducted in a field-practice scenario, we show that sucrosomial iron, an innovative oral iron formulation, is an effective alternative to standard ferrous sulfate in providing iron supplementation in difficult-to-treat populations, such as patients with CD and IDA who are intolerant to iron sulfate. Sucrosomial iron may be particularly suitable for patients with CD and IDA since its innovative formulation does allow good intestinal absorption in patients with inflamed mucosa or gastrointestinal diseases [13].

Fabiano et al. have previously shown that sucrosomial iron has a higher intestinal permeability compared to other iron formulations. Their study showed that supplementation with sucrosomial iron leads to an increased internalization of Fe^3+^ by intestinal Caco-2 cells in vitro, and enhanced Fe^3+^ absorption across rat intestinal epithelium compared to other formulations containing ferric pyrophosphate in ex vivo experimentation. Moreover, this new formulation presents some advantages over the standard ones, such as an increase in ferric iron retention in simulated gastric fluid conditions, a protective action on ferric iron from intestinal enzyme reduction, and promotion of intestinal absorption independently of the DMT-1 carrier [20].

In our study, iron parameters showed, overall, a similar improvement in the two groups, with the wide majority of patients returning to physiological values of the various parameters assessed after 90 days of iron supplementation. Despite this similar outcome it is important to note that patients receiving sucrosomial iron had experienced prior failure with iron sulfate and may, therefore, be considered a more challenging-to-treat population compared to the evaluated controls. Moreover, these patients received a 33% lower dose of iron compared to the control group. Remarkably, with both treatments, increased Hb concentration was also observed in patients with more severe atrophic mucosal damage (MARSH 3a-b-c grade).

No tolerability concerns were reported in either group. However, it is important to notice that sucrosomial iron therapy was associated with less severe gastrointestinal symptoms and enhanced general well-being, as compared to baseline. On the other hand, treatment with iron sulfate was associated with an increased severity of gastrointestinal symptoms through the study period. These findings may be due, at least in part, to the lower dosage of iron required to exert the same effect with the sucrosomial technology as compared to standard supplementation with iron sulfate, [14], but also to the protection of the iron within the phospholipids and sucrester matrix.

Our results are in line with those reported in a recent study where oral sucrosomial iron was compared to intravenous iron supplementation in a group of women of childbearing age undergoing gastric bypass and requiring chronic iron therapy [19]. Ciudin et al. observed that oral sucrosomial iron was as effective as intravenous iron sucrose in increasing iron parameters such as Hb, ferritin, and transferrin saturation after 90 days of treatment. Moreover, no adverse effects were reported with either form of supplementation, thus suggesting that sucrosomial iron represents a safe and effective alternative in patients who require parenteral treatment after bariatric surgery [19].

## 5. Conclusions

We are aware that our study has a number of limitations. Compared to previous studies, our research had an overall short follow-up period and a relatively small sample size, which may result in decreased statistical power, marginal significance for demographic factors, and weaker inference in conclusions. We acknowledge the importance of conducting larger and longer studies on different populations of patients with CD to further evaluate our findings. However, the observed efficacy of sucrosomial iron, together with the lack of associated symptoms in a difficult-to-treat population may have relevance to clinical practice. Indeed, adherence to treatment remains a major determinant of good clinical outcomes in patients with CD who need iron supplementation [24]. We believe that sucrosomial iron holds the potential to increase adherence to iron supplementation in patients with CD and IDA, given the improvement of bothersome gastrointestinal symptoms associated with this therapy.

## Figures and Tables

**Table 1:** Clinical and demographic characteristics of enrolled patients.

Patients’ Characteristics	Sucrosomial Iron Group (*n* = 24)	Iron Sulfate Group (*n* = 19)	*p*
Sex (*n*, male/female)	1/23	1/18	1.00
Age at enrollment (years)	43 ± 10	40 ± 8	0.22
Age at diagnosis (years)	38 ± 8	31 ± 12	0.06
GFD duration (years)	6 ± 5	9 ± 9	0.26
BMI	21 ± 2	22 ± 3	0.34
Smokers, *n* (%)	0 (0%)	2 (16%)	0.08
Menstrual cycle duration (days)	5 ± 2	4 ± 2	0.20
Tampons, *n*	26 ± 17	21 ± 13	0.28
Hp positivity, *n* (%)	2 (8%)	2 (10%)	1.00
Colonoscopy	16	7	0.051
MARSH Grade, *n* (%)	22 (92%)	17 (89%)	0.13
0	7 (32%)	7 (41%)	
1	6 (27%)	2 (12%)	
2	-	1 (6%)	
3a	5 (23%)	5 (29%)	
3b	4 (18%)	-	
3c	-	2 (12%)	
Comorbidities, *n* (%)	9 (37.5%)	5 (26%)	0.44

BMI, body mass index; GFD, gluten-free diet; Hp, *Helicobacter pylori*. Chi-square test and Fisher’s exact test were used for discrete variables; Student’s *t*-test and the Mann-Whitney test were used for continuous variables.

**Table 2:** Blood tests and iron parameters of enrolled patients with CD. Statistical analysis was performed to evaluate the difference before and after therapy (P_(T0–T90)_) and between the two types of iron supplementation at the end of the treatment (P_(groups)_).

	Sucrosomial Iron Group (*n* = 23)				Iron Sulfate Group (*n* = 17)				
	T0	T45	T90	P_(T0–T90)_	T0	T45	T90	P_(T0–T90)_	P_(groups)_
Hemoglobin (g/dL)	10.9 ± 0.7	11.7 ± 0.7	12.0 ± 0.7	0.03	11.0 ± 1.0	12.2 ± 1.0	12.9 ± 1.1	0.002	0.03
MCV (fL)	76.4 ± 5	81.1 ± 5.2	83.5 ± 5.0	0.002	74.2 ± 7.5	81.8 ± 7.3	83.7 ± 6.8	0.008	0.33
Hematocrit (%)	34.2 ± 2.5	36.2 ± 2.9	36.7 ± 3.0	0.08	35.4 ± 3.9	37.6 ± 2.6	38.8 ± 2.6	0.008	0.06
Serum Iron (µg/dL)	41.8 ± 24.0	59.0 ± 29.0	59.9 ± 35.4	0.8	44.9 ± 29.9	66.2 ± 28.0	73.2 ± 21.9	0.14	0.28
Ferritin (ng/mL)	10.7 ± 12.4	16.2 ± 15.2	18.2 ± 15.7	0.04	13.4 ± 15.8	51.2 ± 82.4	59.1 ± 76.3	0.007	0.0001
Transferrin (mg/dL)	311.5 ± 51.3	302.4 ± 46.6	292.1 ± 51.0	0.007	320.6 ± 50.5	293.0 ± 55.4	271.6 ± 40.7	0.006	0.2
Transferrin sat. (%)	10.0 ± 6.4	14.8 ± 9.0	14.8 ± 8.2	0.8	10.6 ± 8.4	16.8 ± 8.0	19.6 ± 6.6	0.03	0.04

MCV, mean corpuscular volume. Chi-square test and Fisher’s exact test were used for discrete variables; Student’s *t*-test and the Mann-Whitney test were used for continuous variables.

**Table 3:** Gastrointestinal symptoms.

	Sucrosomial Iron Group (*n* = 23)				Iron Sulfate Group (*n* = 17)				
	T0	T45	T90	P_(T0–T90)_	T0	T45	T90	P_(T0–T90)_	P_(groups)_
Abdominal Pain	1.9 ± 2.1	2.2 ± 2.2	2.2 ± 2.7	0.07	1.9 ± 1.6	2.7 ± 2.8	2.9 ± 2.5	0.07	0.52
Epigastric Pain	1.8 ± 2.4	1.6 ± 2.4	1.3 ± 1.7	0.7	1.9 ± 2.1	2.4 ± 2.3	2.3 ± 1.8	0.7	0.31
Abdominal Bloating	4.9 ± 3.1	4.4 ± 3.2	4.2 ± 3.5	0.9	3.7 ± 2.5	4.2 ± 3.1	4.1 ± 2.9	0.9	0.6
Diarrhea	1.1 ± 1.6	2.3 ± 2.7	2.0 ± 2.3	0.12	1.5 ± 1.3	2.4 ± 2.0	2.3 ± 1.6	0.11	0.57
Constipation	2.6 ± 3.0	2.0 ± 2.6	1.9 ± 2.8	0.5	2.0 ± 2.2	2.2 ± 2.5	2.8 ± 2.6	0.7	0.8
